# Enterococcus faecalis CRISPR-Cas Is a Robust Barrier to Conjugative Antibiotic Resistance Dissemination in the Murine Intestine

**DOI:** 10.1128/mSphere.00464-19

**Published:** 2019-07-24

**Authors:** Valerie J. Price, Sara W. McBride, Karthik Hullahalli, Anushila Chatterjee, Breck A. Duerkop, Kelli L. Palmer

**Affiliations:** a Department of Biological Sciences, The University of Texas at Dallas, Richardson, Texas, USA; b Department of Immunology & Microbiology, The University of Colorado School of Medicine, Aurora, Colorado, USA; University of Iowa

**Keywords:** CRISPR-Cas, *Enterococcus faecalis*, antibiotic resistance, intestinal colonization, plasmids

## Abstract

CRISPR-Cas systems are barriers to horizontal gene transfer (HGT) in bacteria. Little is known about CRISPR-Cas interactions with conjugative plasmids, and studies investigating CRISPR-Cas/plasmid interactions in *in vivo* models relevant to infectious disease are lacking. These are significant gaps in knowledge because conjugative plasmids disseminate antibiotic resistance genes among pathogens *in vivo*, and it is essential to identify strategies to reduce the spread of these elements. We use enterococci as models to understand the interactions of CRISPR-Cas with conjugative plasmids. Enterococcus faecalis is a native colonizer of the mammalian intestine and harbors pheromone-responsive plasmids (PRPs). PRPs mediate inter- and intraspecies transfer of antibiotic resistance genes. We assessed E. faecalis CRISPR-Cas anti-PRP activity in the mouse intestine and under different *in vitro* conditions. We observed striking differences in CRISPR-Cas efficiency *in vitro* versus *in vivo*. With few exceptions, CRISPR-Cas blocked intestinal PRP dissemination, while *in vitro*, the PRP frequently escaped CRISPR-Cas defense. Our results further the understanding of CRISPR-Cas biology by demonstrating that standard *in vitro* experiments do not adequately model the *in vivo* antiplasmid activity of CRISPR-Cas. Additionally, our work identifies several variables that impact the apparent *in vitro* antiplasmid activity of CRISPR-Cas, including planktonic versus biofilm settings, different donor-to-recipient ratios, production of a plasmid-encoded bacteriocin, and the time point at which matings are sampled. Our results are clinically significant because they demonstrate that barriers to HGT encoded by normal (healthy) human microbiota can have significant impacts on *in vivo* antibiotic resistance dissemination.

**IMPORTANCE** CRISPR-Cas is a type of immune system in bacteria that is hypothesized to be a natural impediment to the spread of antibiotic resistance genes. In this study, we directly assessed the impact of CRISPR-Cas on antibiotic resistance dissemination in the mammalian intestine and under different *in vitro* conditions. We observed a robust effect of CRISPR-Cas on *in vivo* but not *in vitro* dissemination of antibiotic resistance plasmids in the native mammalian intestinal colonizer Enterococcus faecalis. We conclude that standard *in vitro* experiments currently do not appropriately model the *in vivo* conditions where antibiotic resistance dissemination occurs between E. faecalis strains in the intestine. Moreover, our results demonstrate that CRISPR-Cas present in native members of the mammalian intestinal microbiota can block the spread of antibiotic resistance plasmids.

## Abstract (precis)

CRISPR-Cas is a type of immune system in bacteria that is hypothesized to be a natural impediment to the spread of antibiotic resistance genes. In this study, we directly assessed the impact of CRISPR-Cas on antibiotic resistance dissemination in the mammalian intestine and under different *in vitro* conditions. We observed a robust effect of CRISPR-Cas on *in vivo* but not *in vitro* dissemination of antibiotic resistance plasmids in the native mammalian intestinal colonizer Enterococcus faecalis. We conclude that standard *in vitro* experiments currently do not appropriately model the *in vivo* conditions where antibiotic resistance dissemination occurs between E. faecalis strains in the intestine. Moreover, our results demonstrate that CRISPR-Cas present in native members of the mammalian intestinal microbiota can block the spread of antibiotic resistance plasmids.

## INTRODUCTION

CRISPR-Cas systems confer adaptive immunity against mobile genetic elements (MGEs) in bacteria (1–3). CRISPR-Cas systems utilize nucleases programmed with small RNAs to direct sequence-specific cleavage of nucleic acids, including phage and plasmids (4). Most experimental studies of native CRISPR-Cas systems have examined either antiphage defense or defense against electrotransformed plasmids in low-complexity *in vitro* systems. Comparatively little information is available on the roles of CRISPR-Cas in regulating plasmid conjugation, though ∼10% of identified CRISPR protospacers target plasmids (5), and there have been few experimental studies assessing the function of CRISPR-Cas systems within the native ecology of microbial communities. These are major weaknesses in the field from a public health perspective. Conjugative plasmids disseminate antibiotic resistance genes, and CRISPR-Cas systems are naturally occurring barriers that could impede the dissemination of these genes in mammalian microbiota.

We use Enterococcus faecalis as a model organism to study the interactions of CRISPR-Cas systems with conjugative plasmids. E. faecalis is a Gram-positive bacterium, a native inhabitant of the mammalian intestine (6), and an opportunistic pathogen that is among the leading causes of hospital-acquired infections (HAIs) in the United States (7, 8). E. faecalis strains causing HAIs possess unique characteristics relative to strains that normally colonize the human intestine. HAI strains typically have larger genomes resulting from rampant plasmid, phage, and other MGE acquisition (9, 10). Multidrug-resistant (MDR) E. faecalis strains generally lack CRISPR-Cas systems, and there is a correlation between the absence of CRISPR-Cas and the presence of horizontally acquired antibiotic resistance in E. faecalis clinical isolates (11). From genomic analyses, it appears that CRISPR-Cas is a potent barrier to the horizontal acquisition of antibiotic resistance in E. faecalis. Our subsequent efforts have attempted to experimentally address this hypothesis.

The model plasmids we use for our studies are the pheromone-responsive plasmids (PRPs). The PRPs appear to be highly coevolved with E. faecalis (12, 13). PRPs are large (can be >60 kb) and encode accessory traits such as antibiotic resistance, bacteriocin production, reduced UV light susceptibility, and enhanced biofilm formation (12). PRPs encoding antibiotic resistance genes are often present in E. faecalis infection isolates (12, 14–16). The model PRP, pAD1, carries genes for production and self-immunity to a bacteriocin called cytolysin (17). Cytolysin is a lantibiotic-like antimicrobial peptide and hemolysin with activity against a number of Gram-positive bacteria (18, 19).

In this study, we utilize E. faecalis T11RF, a non-MDR strain that encodes a type II CRISPR-Cas system referred to as CRISPR3-Cas (11, 20). Type II CRISPR-Cas systems employ a Cas9-crRNA-tracrRNA (crRNA is CRISPR RNA, and tracrRNA is transactivating crRNA) ribonucleoprotein complex to generate double-stranded DNA breaks in invading MGEs (3, 21, 22). Sequence specificity in the cleavage event is conferred by the crRNA (23). A crRNA is encoded by a short sequence referred to as a spacer, which is derived from and is complementary to a previously encountered MGE (1, 24, 25). The E. faecalis T11RF CRISPR3-Cas system encodes a spacer with perfect sequence complementarity to the *repB* gene of the PRP pAD1 (11, 20). In previous studies, we demonstrated that the E. faecalis CRISPR3-Cas system interferes with the conjugative acquisition of pAM714 (20), a pAD1 variant with an insertion of Tn*917* carrying *ermB* (26, 27). More specifically, pAM714 acquisition is decreased by ∼80-fold in E. faecalis T11RF relative to T11RFΔ*cas9* after 18-h biofilm mating on an agar surface, and CRISPR3-Cas defense against pAM714 requires the targeting spacer (20). These results support our overarching hypothesis that CRISPR-Cas is a significant barrier to the horizontal acquisition of antibiotic resistance in E. faecalis. However, the magnitude of CRISPR3-Cas impact on pAM714 acquisition, while significant, was low compared with the overall high transfer rate of pAM714 under the conditions tested. Many pAM714 molecules escaped CRISPR3-Cas defense despite T11RF possessing functional CRISPR-Cas. We have made similar observations in other E. faecalis strains using both native and engineered CRISPR-Cas systems and with both naturally occurring and engineered plasmids (20, 28, 29). We previously defined the ability of cells to acquire CRISPR-targeted plasmids at high frequencies as CRISPR tolerance (28).

To investigate potential explanations for the seeming discrepancy between the presence of CRISPR-Cas in wild E. faecalis isolates and our *in vitro* observations of statistically significant but middling population-level impact of CRISPR-Cas on conjugative plasmid transfer, we first assessed whether different *in vitro* mating conditions alter conclusions reached about CRISPR-Cas defense efficiency. We compared pAM714 acquisition by wild-type and Δ*cas9* T11RF recipients in planktonic and agar plate biofilm matings using time course experiments and two different initial donor-to-recipient ratios. We performed the same experiments with the PRP pAM771, which is a pAD1 derivative possessing a Tn*917* insertion in the cytolysin locus (26, 30, 31). We reasoned that killing of plasmid-free recipient cells by the cytolysin could “punish” cells that utilize CRISPR-Cas against the plasmid, potentially altering the apparent efficacy of CRISPR-Cas. We also assessed the transfer of pAM714 and pAM771 to wild-type and Δ*cas9* T11RF recipients in a murine intestinal colonization model. We discovered that CRISPR-Cas is a strikingly robust barrier to pAM714 and pAM771 acquisition in the murine intestine.

## RESULTS

### Mating conditions impact CRISPR-Cas activity against pAM714.

**FIG 1:**
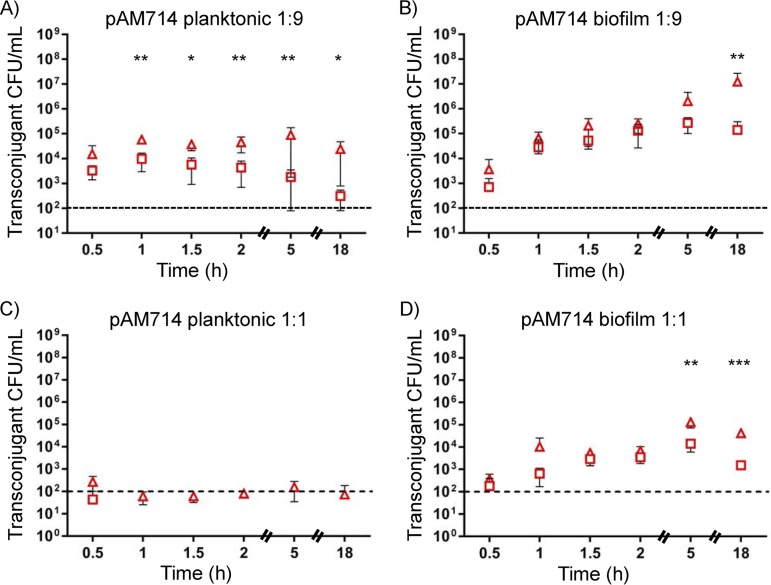
Impact of CRISPR-Cas on pAM714 transconjugant yields under different *in vitro* conditions. The number of transconjugant CFU per milliliter obtained in mating reactions sampled over an 18-h period is shown for E. faecalis T11RF (squares) and T11RFΔ*cas9* (triangles) recipient strains. (A to D) Conjugation was performed under planktonic conditions in broth (A and C) and biofilm conditions on an agar plate (B and D) utilizing E. faecalis OG1SSp as a donor strain. Conjugation reactions were initiated with a 1:9 (A and B) or 1:1 (C and D) donor-to-recipient ratio. The limit of detection is indicated by the dashed lines. Data shown are the averages ± standard deviations (error bars) from a minimum of three independent trials for each time point. Statistical significance was assessed using a two-tailed Student *t* test; values that are significantly different are indicated by asterisks as follows: *, *P* < 0.05; **, *P* < 0.01; ***, *P* < 0.001.

**FIG 2:**
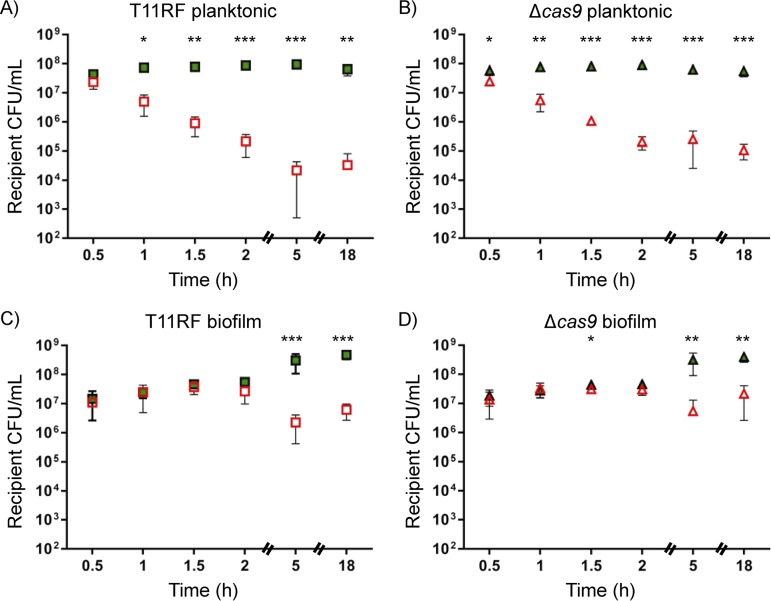
Recipient cell densities for *in vitro* conjugations at a 1:9 donor-to-recipient ratio. (A to D) E. faecalis T11RF (squares) and T11RFΔ*cas9* (triangles) recipient cell densities in CFU/milliliter were determined for both planktonic (A and B) and biofilm (C and D) mating reactions with pAM714 (open red symbols) and pAM771 (closed green symbols) donors. The limit of detection was 10^2^ CFU/ml. Data shown are the averages ± standard deviations from a minimum of three independent trials for each time point for both mating conditions. Statistical significance was assessed using a two-tailed Student *t* test and indicated as follows: *, *P* < 0.05; **, *P* < 0.01; ***, *P* < 0.001.

**FIG 3:**
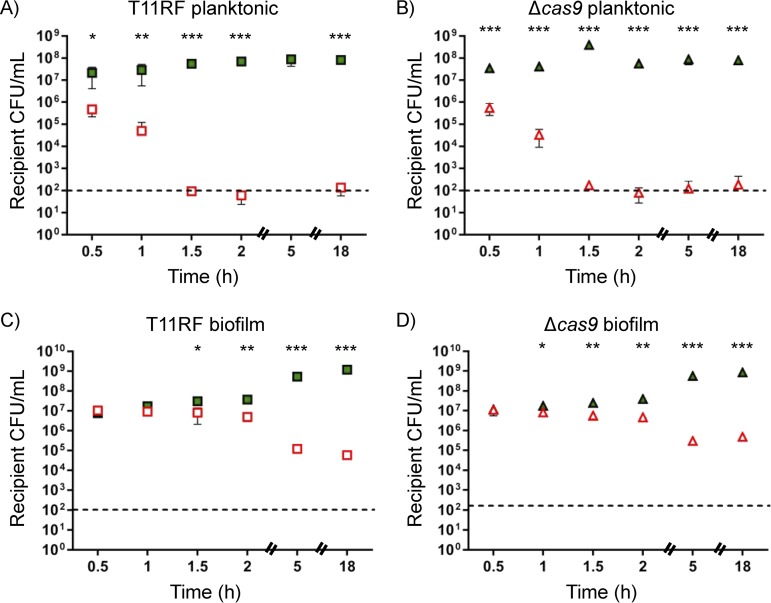
Recipient cell densities for *in vitro* conjugations at a 1:1 donor-to-recipient ratio. (A to D) E. faecalis T11RF (squares) and T11RFΔ*cas9* (triangles) recipient cell densities in CFU/milliliter were determined for both planktonic (A and B) and biofilm (C and D) mating reactions with pAM714 (open red symbols) and pAM771 (closed green symbols) donors. The limit of detection is indicated by the dashed lines. Data shown are the averages ± standard deviations from a minimum of three independent trials for each time point for both mating conditions. Statistical significance was assessed using a two-tailed Student *t* test and indicated as follows: *, *P* < 0.05; **, *P* < 0.01; ***, *P* < 0.001.

We analyzed planktonic and agar plate mating reactions between E. faecalis OG1SSp(pAM714) donors and T11RF or T11RFΔ*cas9* recipients over an 18-h period (Fig. 1; see Table 1 for strain details). We inoculated mating reactions at donor-to-recipient ratios of 1:9 and 1:1 (Fig. 1). Donors were quantified by plating matings on media with spectinomycin, streptomycin, and erythromycin (see Fig. S1 in the supplemental material). Transconjugants were quantified by plating matings on media with rifampin, fusidic acid, and erythromycin (Fig. 1), and total recipients (which includes transconjugants) were quantified by plating matings on media with rifampin and fusidic acid (Fig. 2 and 3). In our experiments, we used erythromycin resistance to track pAM714 conjugation. The *ermB* gene is carried on Tn*917*, which theoretically could transpose from pAM714 into the E. faecalis chromosome, thereby unlinking erythromycin resistance from pAM714 presence. However, Tn*917* transposition frequencies are very low (10^−6^) in the absence of the inducer erythromycin (32). No mating reactions in our study contained erythromycin.

**TABLE 1:** E. faecalis strains used in this study

Strain	Description	Reference(s)
T11RF	Rifampin-fusidic acid-resistant derivative of strain T11	19, 37
T11RFΔ*cas9*	T11RF with an in-frame deletion of *cas9*	19
OG1SSp(pAM714)	Spectinomycin-streptomycin-resistant derivative of strain OG1 harboring pAM714, conferring erythromycin resistance via Tn*917* insertion upstream of the *par* locus; *cyl^+^*	25, 26
OG1SSp(pAM771)	Spectinomycin-streptomycin-resistant derivative of strain OG1 harboring pAM771, conferring erythromycin resistance via Tn*917* insertion disrupting *cylL* of the cytolysin operon; *cyl* mutant	25, 29, 30

10.1128/mSphere.00464-19.1 FIG S1 Donor cell densities for *in vitro* conjugations at a 1:9 donor-to-recipient ratio. E. faecalis T11RF (squares) and T11RFΔ*cas9* (triangles) donor cell densities in CFU/ml were determined for both planktonic (A and B) and biofilm (C and D) mating reactions with pAM714 (open red symbols) and pAM771 (closed green symbols) donors. The limit of detection was 10^2^ CFU/ml. Data shown are the averages and standard deviations from a minimum of three independent trials for each time point for both mating conditions. Statistical significance was assessed using a two-tailed Student *t* test and indicated as follows: *, *P* < 0.05; **, *P* < 0.01; ***, *P* < 0.001. Download FIG S1, PDF file, 0.7 MB. Copyright © 2019 Price et al. 2019 Price et al. This content is distributed under the terms of the Creative Commons Attribution 4.0 International license.

For 1:9 donor-to-recipient ratio experiments, we observed ∼10^3^ to 10^4^ transconjugants for both T11RF and Δ*cas9* recipients after 30 min of mating (Fig. 1A and B). T11Δ*cas9*(pAM714) transconjugant numbers remained stable for the remainder of the planktonic mating experiment, while T11RF(pAM714) transconjugant numbers decreased (Fig. 1A). In contrast, pAM714 transconjugant yields in biofilm matings rose over time for both T11RF and Δ*cas9* recipients, up to the 2-h time point. After that, T11RF(pAM714) transconjugant numbers did not increase further, while T11RFΔ*cas9*(pAM714) transconjugants increased by 2 log units (Fig. 1B). For both planktonic and biofilm matings, we observed significant differences in transconjugant yields between T11RF and Δ*cas9* recipients at the experiment endpoint (18 h) and for some earlier time points. We note that, despite CRISPR-Cas activity, ∼10^5^ pAM714 transconjugants were still observed for T11RF recipients in biofilms (Fig. 1B).

We next assessed conjugation using an equal (1:1) donor/recipient ratio. Increasing donor densities relative to recipients reduces pheromone detection by pheromone-responsive plasmids (33). Transconjugant numbers were overall lower than those observed for 1:9 ratio experiments (Fig. 1C and D). For planktonic matings, transconjugant numbers were at or below our limit of detection; therefore, the impact of *cas9* on transconjugant yield could not be assessed (Fig. 1C). Transconjugants were detected for biofilm matings (Fig. 1D), but the yields were lower than those observed for 1:9 ratio experiments (Fig. 1B). Nevertheless, *cas9* protected recipients from pAM714 acquisition at the 5-h and 18-h time points (Fig. 1D).

### Cytolysin activity depletes recipient cells irrespective of functional CRISPR-Cas.

We hypothesized that the cytolysin encoded by pAM714 could kill recipient cells that utilize CRISPR-Cas against the plasmid. pAM771 is isogenic with pAM714, except that the Tn*917* insertion disrupts *cylL* of the cytolysin biosynthesis gene cluster (26, 30, 31). pAM714 and pAM771 have been utilized in previous studies assessing the impact of cytolysin on virulence, hamster intestinal colonization, and plasmid transfer (30, 31, 34). We performed planktonic and biofilm mating reactions with E. faecalis OG1SSp(pAM771) donors and compared the results with the OG1SSp(pAM714) mating experiments.

Recipient (Fig. 2 and 3) but not donor (Fig. S1 and S2) densities were substantially impacted in all pAM714 mating reactions, irrespective of the presence or absence of *cas9*. The effect was stronger in planktonic matings (Fig. 2A and B and Fig. 3A and B) than in biofilm matings (Fig. 2C and D and Fig. 3C and D), and it was strongest in planktonic matings at a 1:1 donor/recipient ratio, where recipient numbers fell to below the limit of detection after 1.5 h of mating (Fig. 3A and B). These results are consistent with pAM714 transconjugant yields under these conditions (Fig. 1C). In biofilm matings, striking effects on recipient cell densities were not observed until later time points (5 h and 18 h; Fig. 2C and D and Fig. 3C and D).

10.1128/mSphere.00464-19.2 FIG S2 Donor cell densities for *in vitro* conjugations at a 1:1 donor-to-recipient ratio. E. faecalis T11RF (squares) and T11RFΔ*cas9* (triangles) donor cell densities in CFU/ml were determined for both planktonic (A and B) and biofilm (C and D) mating reactions with pAM714 (open red symbols) and pAM771 (closed green symbols) donors. The limit of detection was 10^2^ CFU/ml. Data shown are the averages and standard deviations from a minimum of three independent trials for each time point for both mating conditions. Statistical significance was assessed using a two-tailed Student *t* test and indicated as follows: *, *P* < 0.05; **, *P* < 0.01; ***, *P* < 0.001. Download FIG S2, PDF file, 0.7 MB. Copyright © 2019 Price et al. 2019 Price et al. This content is distributed under the terms of the Creative Commons Attribution 4.0 International license.

**FIG 4:**
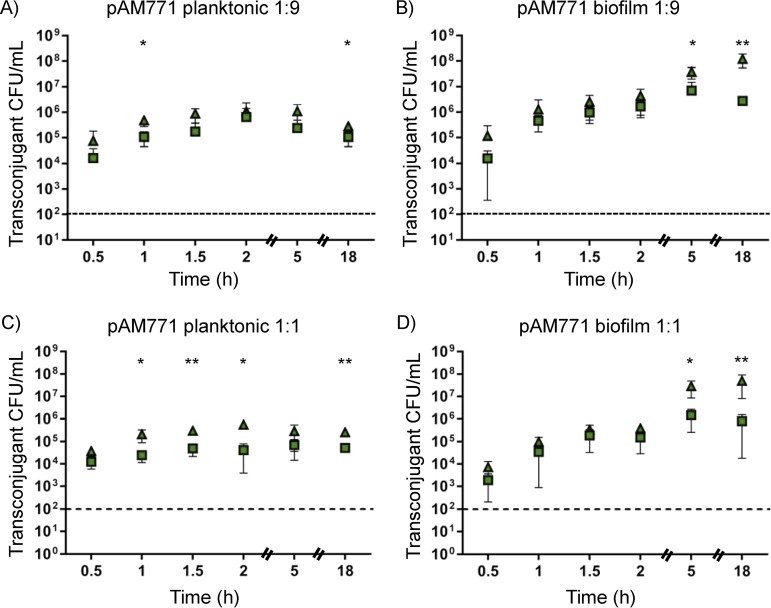
Impact of CRISPR-Cas on pAM771 transconjugant yields under different *in vitro* conditions. The number of transconjugant CFU/milliliter obtained in mating reactions sampled over an 18-h period is shown for E. faecalis T11RF (squares) and T11RFΔ*cas9* (triangles) recipient strains. (A to D) Conjugation was performed under planktonic conditions in broth (A and C) and biofilm conditions on an agar plate (B and D) utilizing E. faecalis OG1SSp as a donor strain. Conjugation reactions were initiated with a 1:9 (A and B) or 1:1 (C and D) donor-to-recipient ratio. The limit of detection is indicated by the dashed lines. Data shown are the averages ± standard deviations from a minimum of three independent trials for each time point. Statistical significance was assessed using a two-tailed Student *t* test and indicated as follows: *, *P* < 0.05; **, *P* < 0.01; ***, *P* < 0.001.

Unlike observations from pAM714 matings, recipient numbers were stably high in pAM771 matings. Moreover, pAM771 transconjugant yields were not substantially impacted by the donor/recipient ratio (Fig. 4). Similar transconjugant yields were detected for planktonic matings at 1:9 (Fig. 4A) and 1:1 (Fig. 4C) ratios and for biofilm matings at the two ratios (Fig. 4B and D, respectively). The effect of *cas9* was minor in magnitude but statistically significant at the end of planktonic mating. Deletion of *cas9* increased plasmid acquisition significantly, by ∼2 log units, after 18 h of biofilm mating.

Overall, our results with *in vitro* experiments demonstrate that planktonic versus biofilm settings, different donor/recipient ratios, production of a plasmid-encoded bacteriocin, and the time points at which matings are sampled all impact transconjugant yields and conclusions reached about the apparent activity of CRISPR-Cas. Moreover, CRISPR tolerance is consistently observed *in vitro*, with the exception of settings where little plasmid transfer occurs into any recipient (pAM714 planktonic matings at a 1:1 donor/recipient ratio; Fig. 1C).

### CRISPR-Cas is a robust barrier to PRP acquisition in the murine intestine.

**FIG 5:**
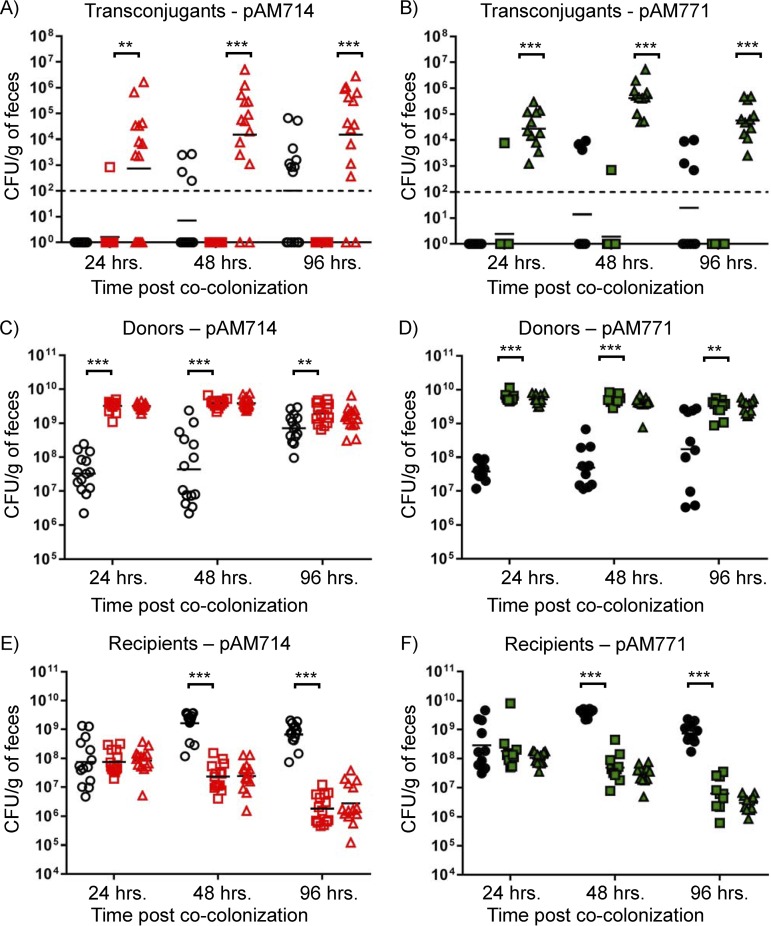
Impact of CRISPR-Cas on plasmid transfer in the mouse intestine. (A to F) The numbers of transconjugant (A and B), donor (C and D), and recipient (E and F) CFU/g of feces for individual mice were determined by plating feces on selective agars. Each symbol represents the value for one mouse. Experimental groups are described in Materials and Methods. Black horizontal bars represent the geometric means of data for each group. (A to F) Data for *in vivo* pAM714 (A, C, and E; open red symbols) and pAM771 (B, D, and F; closed green symbols) transfer are shown. Control mice cocolonized by E. faecalis OG1SSp and T11RF are represented by open circles. Mice colonized with T11RF recipients are represented by squares. Mice colonized with T11RFΔ*cas9* recipients are represented by triangles. The limit of detection is indicated by the dashed line. Statistical significance was assessed using a two-tailed Student *t* test. Values that are significantly different are indicated by bars and asterisks as follows: **, *P* < 0.01; ***. *P* < 0.001.

We assessed CRISPR3-Cas activity against pAM714 and pAM771 in a mouse model of E. faecalis intestinal dysbiosis. To establish antibiotic-induced dysbiosis, mice were administered a cocktail of antibiotics in their drinking water for 7 days, followed by regular drinking water for 24 h. The mice were colonized sequentially with recipient and donor E. faecalis strains at a 1:1 donor/recipient ratio. Fecal pellets were collected at 24, 48, and 96 h after cocolonization and homogenized, and the numbers of transconjugants, donors, and recipients were determined (Fig. 5). Experimental groups consisting of different combinations of donor and recipient strains were used: OG1SSp with T11RF as a plasmid-free control group, E. faecalis OG1SSp(pAM714) donors with T11RF recipients, and OG1SSp(pAM714) donors with T11RFΔ*cas9* recipients. In separate experiments, OG1SSp(pAM771) donors were used.

We detected pAM714 transconjugants in only 1 mouse of the 14 mice colonized with T11RF recipients at 24 h after cocolonization, and for none of the mice at 48-h and 96-h time points (Fig. 5A). Strikingly, pAM714 transconjugants at densities up to ∼5 × 10^6^ CFU/g of feces were observed for 12 of 14 mice colonized with T11RFΔ*cas9* recipients over the course of the experiment (Fig. 5A). We screened 36 presumptive E. faecalis T11RFΔ*cas9*(pAM714) transconjugants by PCR amplification of the pAM714 *repB* gene; all possessed this gene (Fig. S3). We observed that some control mice (no plasmid) at 48- and 96-h time points had colony growth on media with selection for transconjugants (i.e., media supplemented with rifampin, fusidic acid, and erythromycin) (Fig. 5A). We screened 20 of these colonies by PCR amplification of the pAM714 *repB* gene; none possessed this gene (Fig. S3). We infer that recipients received erythromycin resistance determinants from the native mouse microbiota via a non-pAM714-dependent mechanism.

10.1128/mSphere.00464-19.3 FIG S3 PCR confirms that experimental transconjugants carry pAM714 and spontaneous resistant isolates from control animals do not. Agarose gels show PCR amplification products for the *repB* gene of pAM714 at 24 (A), 48 (B), and 96 (C) hours postcolonization, and the lack of a *repB* amplification signal in isolates originating from control mice and growing on transconjugant selection agar (B and D). PCR reactions for the presence and absence of the *repB* gene using E. faecalis strains OG1SSp(pAM714), OG1SSp, T11RF, and T11RFΔ*cas9* are included on each gel. Rif, rifampin; Fus, fusidic acid; Erm, erythromycin. Download FIG S3, PDF file, 1.1 MB. Copyright © 2019 Price et al. 2019 Price et al. This content is distributed under the terms of the Creative Commons Attribution 4.0 International license.

We performed identical *in vivo* conjugation experiments with E. faecalis OG1SSp(pAM771) donors. Fewer mice were observed with sporadic erythromycin resistance in the control group for pAM771 experiments (Fig. 5B). T11RF(pAM771) transconjugants were detected for only 1 of 10 mice at each of the 24- and 48-h time points, whereas T11RFΔ*cas9*(pAM771) transconjugants were detected in all 11 mice and at all time points (Fig. 5B).

Overall, these data demonstrate that there is a profound impact of CRISPR-Cas on plasmid transfer between E. faecalis strains in the dysbiotic mouse intestine. These observations are in contrast to any *in vitro* condition evaluated, where either plasmid transfer was not observed (for 1:1 ratio planktonic matings) or transconjugants arose despite recipients having CRISPR-Cas defense. Moreover, cytolysin did not impact *in vivo* plasmid transfer, as was observed for *in vitro* transfer. This is consistent with a previous study that analyzed transfer of pAM714 and pAM771 between E. faecalis in the hamster intestinal tract (30).

### Potential cytolysin-independent *in vivo* colonization benefit to strains possessing a PRP.

We next assessed whether cytolysin impacted the colonization of E. faecalis donors in the mouse intestine. We compared donor densities in control mice colonized with E. faecalis OG1SSp to those colonized with OG1SSp(pAM714) (Fig. 5C) or OG1SSp(pAM771) (Fig. 5D). We observed no benefit to donors possessing pAM714 versus pAM771. However, donor densities in the control group were significantly reduced compared to plasmid-bearing donors at all time points. These data suggest that there is a cytolysin-independent colonization benefit for OG1SSp harboring pAM714 or pAM771. This is consistent with recent observations for E. faecalis harboring the PRP pCF10 during intestinal colonization of germfree mice (35).

At 24 h after cocolonization, recipient strain densities from control, pAM714, and pAM771 test groups were similar (Fig. 5E and F). On average, E. faecalis T11RF recipient densities increased in control mice at subsequent time points but decreased in both pAM714 and pAM771 test groups (Fig. 5E and F). No differences were observed for recipient densities in pAM714 versus pAM771 groups. This demonstrates that the reduction in recipient cell densities observed *in vitro* for pAM714 but not pAM771 matings (Fig. 2 and 3) does not occur in the *in vivo* model tested here. Rather, our data suggest that there is a cytolysin-independent fitness advantage for pAM714/pAM771 donors *in vivo*. We also note that no differences were observed in T11RF and T11RFΔ*cas9* colonization, indicating that the presence or absence of *cas9* does not impact intestinal colonization success in this model (Fig. 5E and F).

## DISCUSSION

We have found that native CRISPR-Cas encoded by a member of the mammalian intestinal microbiota can block the *in vivo* dissemination of an antibiotic resistance plasmid in a murine intestinal colonization model. This is in contrast to *in vitro* observations, where the same plasmid is frequently acquired by recipient cells despite CRISPR-Cas. For the E. faecalis CRISPR1-Cas system we previously investigated (28, 29), these tolerant cells harboring both CRISPR-Cas and a plasmid it targets have an *in vitro* growth defect that is resolved by either plasmid loss or by mutation of CRISPR-Cas when antibiotic selection for the plasmid is applied. We did not detect CRISPR tolerance *in vivo*. One possible explanation for this is that CRISPR-Cas is far more effective *in vivo* than *in vitro*, and transconjugants never arise in cells possessing functional CRISPR-Cas *in vivo*. Another explanation is that they do arise, but their growth defect combined with turnover of intestinal contents results in their rapid elimination *in vivo*. One method to test this would be to add erythromycin selection *in vivo*; we would expect to observe high densities of T11RF plasmid transconjugants that are CRISPR-Cas mutants. We were not able to test this in our current model system because of the erythromycin inducibility of Tn*917*, which would complicate plasmid detection.

What mechanisms underlie our observations about the impact of CRISPR-Cas on conjugative plasmid transfer *in vitro* and *in vivo*? Several factors may factor in this process, including plasmid host range, donor-to-recipient ratios and their relative colonization densities, community spatial structure (i.e., biofilms), flow and dilution rate, nutrient availability, community diversity and the relative densities of plasmid-susceptible versus nonsusceptible hosts, and selection for the plasmid. With the PRPs, there is the additional consideration of pheromone concentration; the pheromone is a short peptide elaborated by recipient E. faecalis cells (and some other bacteria) that induces transcription of conjugation genes in the donor strain (12, 13). Finally, there are CRISPR-Cas-specific factors about which little is known, such as the *in vitro* versus *in vivo* transcriptional and posttranscriptional regulation of *cas9*. We confirmed that several of these factors influence PRP transconjugant yield *in vitro*. Specifically, cytolysin biosynthesis encoded by the plasmid negatively impacted recipient densities, and the donor/recipient ratio, which affects induction of conjugation by pheromone signaling (33), impacted transconjugant yields.

We determined that the pAM714 conjugation frequency to E. faecalis T11RFΔ*cas9* recipients is ∼10^0^ to 10^−2^ transconjugants per donor (TC/D) for *in vitro* broth and agar plate biofilm experiments, while *in vivo*, it ranges from 10^−3^ to 10^−7^ (see Fig. S4 in the supplemental material). The conjugation frequency of PRPs can be modulated by deleting aggregation substance in the plasmid, which should reduce conjugation frequency in broth cultures but not biofilms (12), or by interfering with pheromone production by plasmid-free recipient cells. This will be addressed in future work. Also to be addressed is the effect of changing the total cell count of the donors and recipients at the time of culture inoculation; initiating cultures with fewer cells may more accurately reflect the nature of intestinal colonization by E. faecalis.

10.1128/mSphere.00464-19.4 FIG S4 Frequency of conjugation *in vitro* and in the mouse intestine. Conjugation frequencies for pAM714 in the mouse intestine (A), planktonic at 1:9 donor/recipient ratio (C), and biofilm at 1:9 donor/recipient ratio (E) settings are shown as transconjugants per donor. Conjugation frequencies for pAM771 in the mouse intestine (B), planktonic at 1:9 donor/recipient ratio (D), and biofilm 1:9 donor/recipient ratio (F) settings are also shown. E. faecalis T11RF recipients are represented by squares; T11RFΔ*cas9* recipients are represented as triangles. For calculating *in vivo* conjugation frequencies, the conjugation frequency for each mouse was determined by dividing the transconjugant CFU/g by the donor CFU/g; each symbol represents the value for one mouse. Black horizontal bars represent the geometric mean for data for each group. No symbol means that a frequency could not be calculated because one or both of the values (donor CFU/g or transconjugant CFU/g) were zero. Statistical significance was assessed using a two-tailed Student *t* test and indicated as follows: * , *P* < 0.05; **, *P* < 0.01; ***, *P* < 0.001. Download FIG S4, PDF file, 1.2 MB. Copyright © 2019 Price et al. 2019 Price et al. This content is distributed under the terms of the Creative Commons Attribution 4.0 International license.

In the *in vivo* model used here, we induced intestinal dysbiosis with antibiotics, allowed mice to recover for 1 day, and then colonized them with E. faecalis. This models what can occur in patients after receiving antibiotic therapy. Another mouse model used in the field establishes long-term colonization of E. faecalis without major disruption of normal (healthy) intestinal microbiota (36). Further, a recent study utilized a germfree mouse model to examine *in vivo* transfer of the PRP pCF10 among intestinal E. faecalis (35). In the germfree model, enterococci achieve very high densities, and diversity is very low. In the native colonization model, diversity is high, and production of the Bac-21 bacteriocin from the PRP pPD1 significantly enhances E. faecalis colonization (36). These two models can be used to assess how community diversity and the densities of plasmid-susceptible and nonsusceptible hosts impact CRISPR-Cas efficacy *in vivo*.

How far can we extrapolate from studies with E. faecalis to other members of the mammalian microbiota, and from PRPs to other plasmids with different properties and host ranges? Put another way, does CRISPR-Cas encoded by genes of other members of the native microbiota confer the same robust defense against antibiotic resistance plasmids as observed for E. faecalis and PRPs? Will E. faecalis CRISPR-Cas defense against non-PRP plasmids be equally robust? Much future work remains to elucidate these questions.

## MATERIALS AND METHODS

### Bacteria and reagents used.

Strains used in this study are shown in Table 1. E. faecalis strains were cultured in brain heart infusion (BHI) broth or on BHI agar at 37°C. Antibiotic concentrations used were as follows: rifampin, 50 μg/ml; fusidic acid, 25 μg/ml; spectinomycin, 500 μg/ml; streptomycin, 500 μg/ml; erythromycin, 50 μg/ml. Antibiotics were purchased from Sigma-Aldrich or Research Products International (RPI).

### Conjugation experiments.

Donor and recipient strains were cultured overnight in BHI broth in the absence of antibiotic selection. The following day, cultures were diluted 1:10 into fresh BHI and incubated at 37°C for 1.5 h. For planktonic conjugations at a 1:9 donor/recipient ratio, 2 ml of donor and 18 ml of recipient were mixed in a flask and incubated without agitation at 37°C for 30 min to 18 h. For planktonic conjugations at a 1:1 donor/recipient ratio, 10 ml of donor and 10 ml of recipient were mixed in a flask and incubated without agitation at 37°C for 30 min to 18 h. At each time point, 1 ml of the mating reaction mixture was removed and used for serial dilutions and plating on selective media. For biofilm mating reactions at a 1:9 donor/recipient ratio, 100 μl of donor was mixed with 900 μl of recipient, and for mating reactions at a 1:1 donor/recipient ratio, 500 μl of donor was mixed with 500 μl of recipient. The mixture was centrifuged for 1 min at 16,000 × *g*. After centrifugation, 100 μl supernatant was used to resuspend the pellet, which was then spread-plated on nonselective BHI agar. To allow for sampling of multiple time points of biofilms, multiple identical conjugation reactions were generated using the same donor and recipient inocula. The conjugation reaction mixtures were incubated at 37°C for 30 min to 18 h. At each time point, cells were collected by washing and scraping an agar plate using 2 ml of 1× phosphate-buffered saline (PBS) supplemented with 2 mM EDTA, and serial dilutions were plated on selective media. For all matings, BHI agar supplemented with antibiotics was used to quantify the donor (spectinomycin, streptomycin, and erythromycin), recipient (rifampin and fusidic acid), and transconjugant (rifampin, fusidic acid, and erythromycin) populations. Plates were incubated for 36 to 48 h at 37°C. Plates with 30 to 300 colonies were used to calculate the number of CFU per milliliter.

### Mouse model of E. faecalis colonization.

Seven days prior to bacterial colonization, 6- to 8-week-old C57BL/6 mice were gavaged with 100 μl of an antibiotic cocktail (streptomycin [1 mg/ml], gentamicin [1 mg/ml], erythromycin [200 μg/ml]), and given a water bottle *ad libitum* with the same antibiotic cocktail for 6 days following gavage. Twenty-four hours before bacterial inoculation, antibiotic water was removed and replaced with standard sterile antibiotic-free water. Bacteria were grown overnight in BHI, and mice were gavaged with 10^9^ CFU in PBS of each bacterial strain as experimental groups indicated (1:1 donor/recipient ratio). Samples used for gavage were plated on BHI to confirm that inocula were equal across strains. Fecal samples from mice were collected at 0 h, 24 h, 48 h, and 96 h. Fecal samples were resuspended in 1 ml of sterile PBS, and dilutions were plated on BHI agar supplemented with antibiotics to quantify the donor (spectinomycin, streptomycin, and erythromycin), recipient (rifampin and fusidic acid), and transconjugant (rifampin, fusidic acid, and erythromycin) populations. Plates were incubated for 36 to 48 h at 37°C. Plates with 30 to 300 colonies were used to calculate CFU/gram of feces. Experiments were performed in duplicate or triplicate as follows. For E. faecalis OG1SSp pAM714/T11RF (with or without *cas9*) cocolonization, three independent experiments were performed consisting of 4, 4, and 6 mice per group per experiment. For OG1SSp pAM771/T11RF (with or without *cas9*) cocolonization, two independent experiments were performed consisting of five mice per group, except in the second experiment where five mice were used for each group (control and wild-type T11RF groups) and six mice were used for the T11RFΔ*cas9* group. Data from individual experimental replicates were combined and graphed together. All animal protocols were approved by the Institutional Animal Care and Use Committee of the University of Colorado Anschutz Medical Campus (protocol 00253).

### Colony PCR to verify *in vivo* transconjugants.

Fecal pellets were collected at 0 h, 24 h, 48 h, and 96 h, weighed, and resuspended in 1 ml PBS. Portions (20 μl) were plated at multiple dilutions on BHI containing rifampin, fusidic acid, and erythromycin. Individual colonies were picked and resuspended in 20 μl nuclease-free water, and 1 μl was used in PCR with *Taq* DNA polymerase (New England Biolabs). Primers amplified the *repB* region of plasmids pAM714 and pAM771 (pAD1 *repB*-For [For stands for forward], 5′-CGT TCC ATG TGT CTA ACA ATT GTA TTA AAC-3′, and pAD1 *repB*-Rev [Rev stands for reverse], 5′-CGA TGA TGG TAG CAA TTC AAG AAG G-3′).
