# Supplementary figures and images for: Enterococcus faecalis CRISPR-Cas Is a Robust Barrier to Conjugative Antibiotic Resistance Dissemination in the Murine Intestine
Source: mSphere. 2019 Jul 24;4(4):e00464-19. doi: 10.1128/mSphere.00464-19 (PMC6656873; doi:10.1128/mSphere.00464-19)

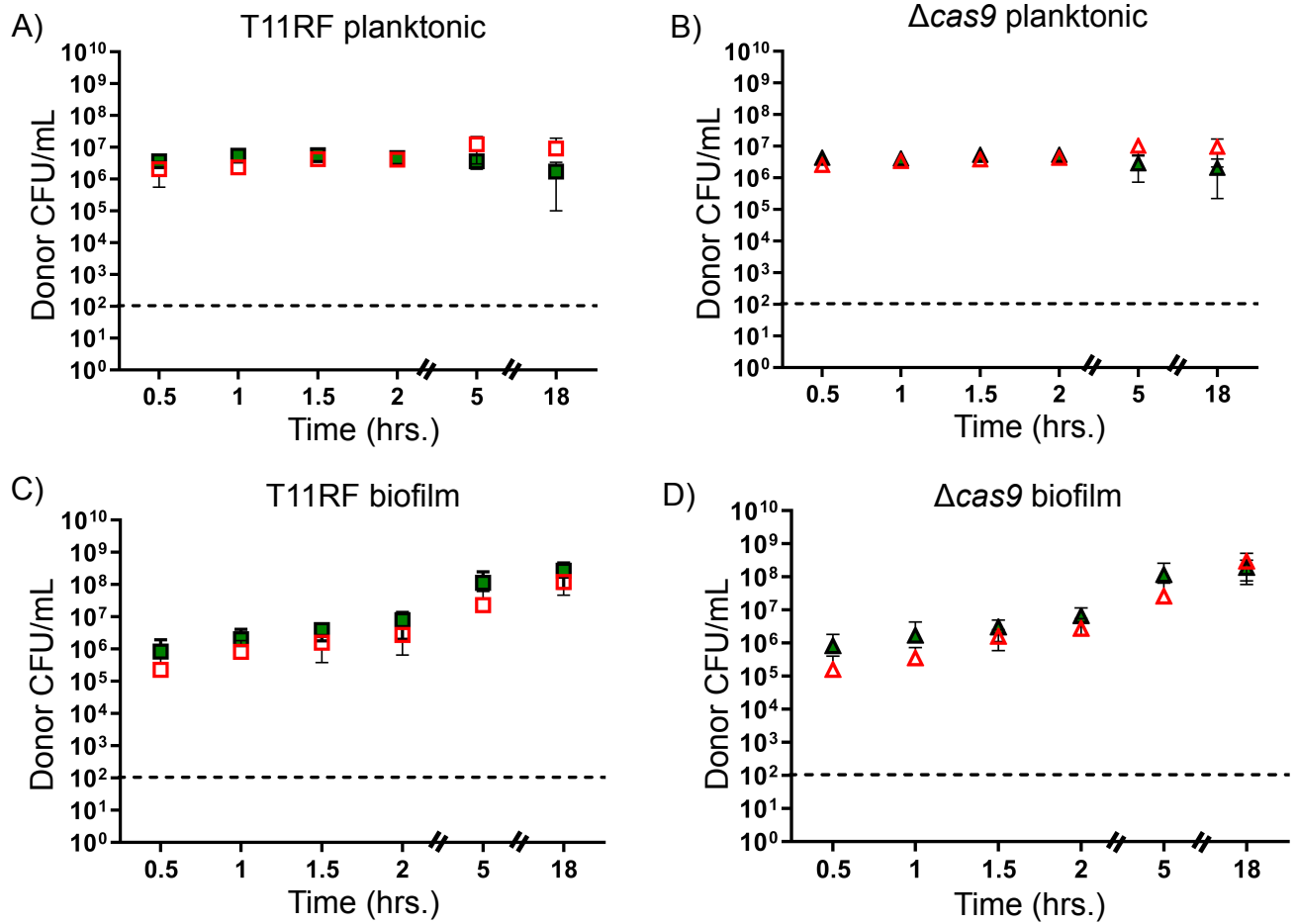

Figure S1

Supplement: FIG S1 [file mSphere.00464-19-sf001.pdf]

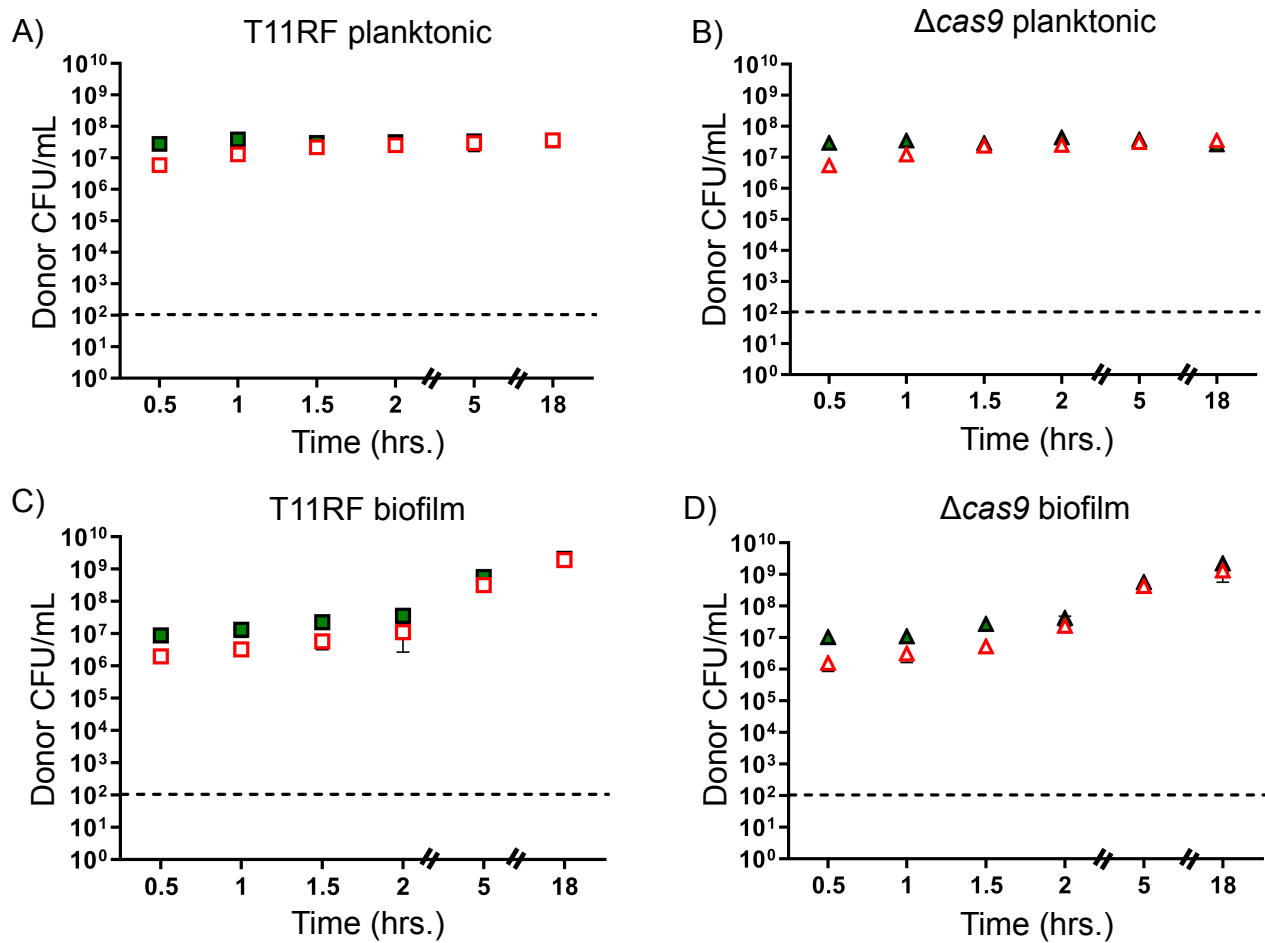

**Figure S2**

Supplement: FIG S2 [file mSphere.00464-19-sf002.pdf]

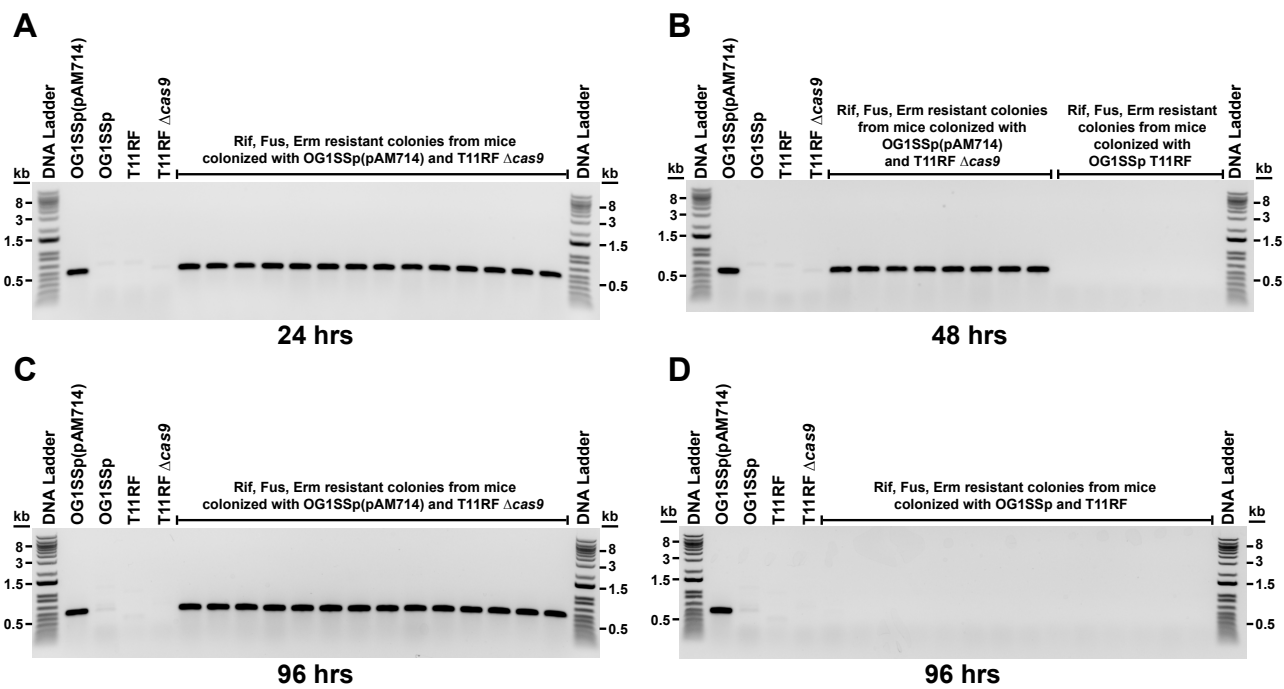

**Figure S3**

Supplement: FIG S3 [file mSphere.00464-19-sf003.pdf]

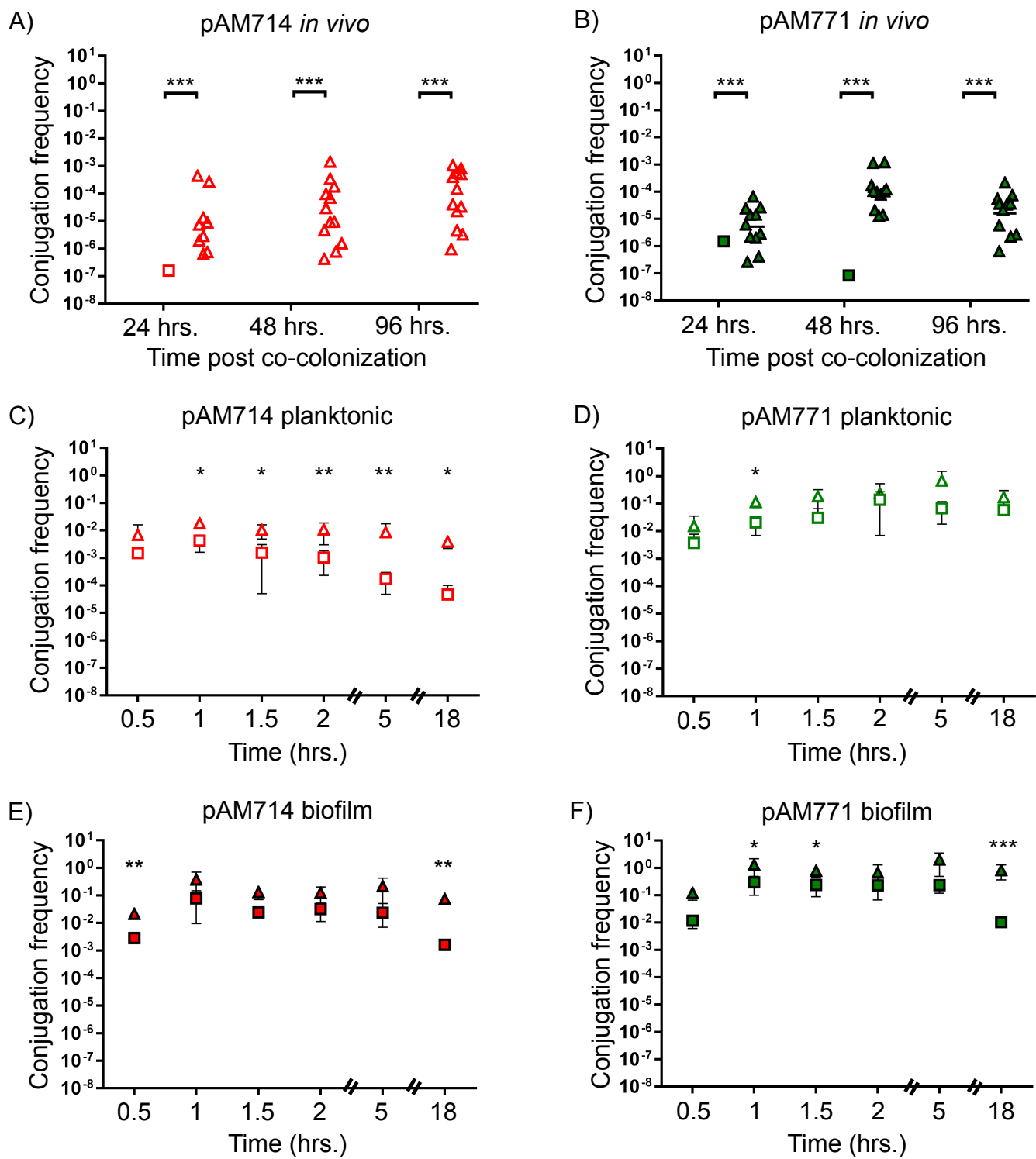

Figure S4

Supplement: FIG S4 [file mSphere.00464-19-sf004.pdf]
